# Abordando as Disparidades de Gênero nos Cuidados Cardiovasculares: Orientações das Diretrizes para ICP em Mulheres e o Enigma de Hua-Mulan

**DOI:** 10.36660/abc.20240824

**Published:** 2025-02-20

**Authors:** Pedro Guimarães Silva, Henrique Barbosa Ribeiro

**Affiliations:** 1 Hospital das Clínicas Faculdade de Medicina Universidade de São Paulo São Paulo SP Brasil Instituto do Coração do Hospital das Clínicas da Faculdade de Medicina da Universidade de São Paulo, São Paulo, SP – Brasil; 2 Hospital Sirio-Libanês São Paulo SP Brasil Hospital Sirio-Libanês, São Paulo, SP – Brasil; 3 Hospital Samaritano Paulista São Paulo SP Brasil Hospital Samaritano Paulista, São Paulo, SP – Brasil

**Keywords:** ntervenção Coronária Percutânea, Equidade de gênero, Doença das Coronárias, Stents

O impacto das doenças cardiovasculares (DCV) não é distribuido uniformemente na população. Ainda que fatores biológicos contribuem para sua prevalência, questões sociais, culturais, econômicas, ambientais e de gênero também são elementos determinantes para a condução e tratamento da doença isquêmica do coração (DIC).^1-3^Apesar dos avanços significativos nos cuidados cardiovasculares e no campo das intervenções coronárias percutâneas (ICP), disparidades entre gêneros ainda persistem nos campos de diagnóstico acesso a recursos e tratamento das DCV.

De acordo com o relatório de 2019 da Global Burden of Disease (GBD) Network^1^as DCV foram responsáveis por 35% de todas as mortes femininas no mundo, com impacto particularmente pronunciado em países em desenvolvimento, onde barreiras socioeconômicas dificultam o acesso a cuidados de saúde adequados.^4^No Brasil, os dados da GBD revelaram que 12% das mortes foram atribuídas à DIC, compreendendo 32,3% do total de mortes relacionadas a DCV. Embora tenha havido melhoras relvantes nos últimos anos, as mulheres continuam a apresentar taxas de mortalidade relacionadas à DIC (29,9%) um pouco maiores em comparação aos homens (27,6%).^5^Essas estatísticas ressaltam a necessidade urgente de estratégias direcionadas para abordar as desigualdades de gênero e os determinantes mais amplos da saúde no tratamento de DCV.^6,7^

Somando-se a essa complexidade está a sub-representação de mulheres em estudos que avaliam os resultados da ICP, deixando os benefícios potenciais para as mulheres pouco explorados e especulativos. Essa falta de representação levanta uma questão inquietante: as artérias coronárias das mulheres estão recebendo a atenção que realmente precisam? Como a lendária Hua Mulan, que se disfarçou de homem para lutar na guerra, as diretrizes atuais da ICP — projetadas principalmente com pacientes do sexo masculino em mente — poderiam ignorar nuances críticas no tratamento de pacientes do sexo feminino? Essa metáfora serve como um lembrete pungente de que uma abordagem generalizada pode fazer que desconsideremos os desafios únicos enfrentados por mulheres com DIC.

Nesta edição da revista, Braga et al.^8^ avaliaram se a ICP atual orientada por diretrizes fornece às mulheres brasileiras os mesmos benefícios que aos seus colegas homens. Realizado em um centro cardiovascular terciário público no Brasil, o estudo analisou os resultados em 1.146 mulheres que foram submetidas a ICP entre 2019 e 2020. A coorte, com idade média de 65 anos, exibiu alta prevalência de fatores de risco cardiovascular tradicionais, como hipertensão (88%), diabetes (47,5%) e dislipidemia (85%). A maioria dos pacientes (69%) foi admitida com síndrome coronariana aguda (SCA). Os procedimentos de ICP foram predominantemente bem-sucedidos, com 97,7% dos pacientes e 98,4% dos vasos tratados alcançando resultados favoráveis. Complicações ocorreram em 14,2% dos pacientes, com uma taxa de mortalidade hospitalar de 1,2%. Infarto do miocárdio periprocedimental foi relatado em 3,6% dos casos. No entanto, a ausência de dados sobre o fenômeno de *slow-flow/no-reflow* — uma complicação associada a piores resultados — limita o escopo da análise. Os preditores de eventos cardíacos e cerebrovasculares adversos graves (MACCE) hospitalares incluíram acidente vascular cerebral prévio, doença renal crônica e insucesso do procedimento, destacando a interação complexa de fatores anatômicos e clínicos que influenciam os resultados nas mulheres.^9^

Apesar desses resultados favoráveis, o estudo deixou de explorar determinantes sociais críticos da saúde, como níveis educacionais e redes de apoio social. Esses fatores podem oferecer insights mais profundos sobre seu impacto na saúde cardiovascular, e sua exploração pode levar a avanços significativos em nossa compreensão e gerenciamento da DCV.

Os dados do estudo, derivados de um ambiente hospitalar público, apresentam desafios únicos, incluindo uma população caracterizada por níveis socioeconômicos e educacionais mais baixos, uso limitado de ferramentas avançadas de imagem intravascular e fisiologia, disponibilidade restrita de terapias antiplaquetárias e limitações baseadas em custos nos tipos e números de stents farmacológicos utilizados. Essas condições do mundo real contrastam fortemente com os cenários idealizados em muitas diretrizes internacionais e ensaios clínicos para o tratamento de doença coronária aguda ou crônica.^10-13^

Ao longo de uma média de 576 dias, os dados de acompanhamento confirmaram ainda mais os benefícios sustentados da ICP, com uma taxa de sobrevida livre de MACCE de 86%, uma mortalidade cardíaca de 3,5% e 8% de SCA recorrente, ressaltando sua eficácia no tratamento de doença arterial coronária aguda e crônica. Os preditores desses eventos incluíram admissão por SCA na ICP índice e, bem como MACCE na fase hospitalar, destacando a importância da intervenção precoce e do tratamento abrangente pós-alta. Esses achados fornecem segurança e esperança para melhorar os resultados de longo prazo para essa população de alto risco.

Finalmente, a resposta é cautelosamente positiva ao abordar o “enigma Hua-Mulan” — se as evidências de ICP derivadas predominantemente de estudos focados em homens podem ser aplicadas a pacientes do sexo feminino. Embora este estudo corrobore um progresso significativo na aplicação das diretrizes atuais para mulheres com doença arterial coronária, ele também enfatiza a importância de adaptar abordagens para levar em conta os perfis de risco exclusivos das mulheres, as especificidades anatômicas e os determinantes sociais da saúde.

Braga et al.^8^ nos lembram contundentemente tanto do progresso feito quanto dos desafios no enfrentamento das disparidades de gênero na saúde cardiovascular. Ao priorizar estratégias focadas em mulheres, os provedores de saúde podem melhorar os resultados para mulheres com doença arterial coronária, garantindo acesso equitativo ao tratamento e aumentando as taxas de sobrevivência. Pesquisa colaborativa, educação e esforços para formulação de políticas públicas serão essenciais para preencher essas lacunas e moldar um futuro em que os resultados da saúde cardiovascular possam ser menos influenciados pelo gênero.
