# Supplementary material for: Abordando as Disparidades de Gênero nos Cuidados Cardiovasculares: Orientações das Diretrizes para ICP em Mulheres e o Enigma de Hua-Mulan
Source: Arq Bras Cardiol. 2025 Feb 20;122(1):e20240824. [Article in Portuguese] doi: 10.36660/abc.20240824 (PMC11870118; doi:10.36660/abc.20240824)
Supplement: Translation

# Translation: Addressing Gender Disparities in Cardiovascular Care: Guideline-Oriented PCI for Women and the Hua-Mulan Conundrum

**DOI:** 10.36660/abc.20240824i

**Authors:** Pedro Guimarães Silva, Henrique Barbosa Ribeiro

**Affiliations:** 1 Brazil Instituto do Coração do Hospital das Clínicas da Faculdade de Medicina da Universidade de São Paulo, São Paulo, SP – Brazil; 2 Brazil Hospital Sirio-Libanês, São Paulo, SP – Brazil; 3 Brazil Hospital Samaritano Paulista, São Paulo, SP – Brazil

**Keywords:** Percutaneous Coronary Intervention, Gender Equity, Coronary Disease, Stents

The burden of cardiovascular disease (CVD) is not evenly distributed across populations. While biological factors contribute to its prevalence, social, cultural, economic, environmental, and gender-related issues also play significant roles in shaping the unfavorable outcomes associated with ischemic heart disease (IHD).^1^ Despite significant advances in cardiovascular care and the advent of modern percutaneous coronary interventions (PCI), gender disparities in the diagnosis, treatment, and outcomes of CVD persist.

According to the Global Burden of Disease (GBD) Network’s 2019 report,^1^ CVD accounted for 35% of all female deaths globally, with the impact particularly pronounced in developing countries, where socioeconomic barriers hinder access to adequate healthcare.^4^ In Brazil, GBD data revealed that 12% of deaths were attributed to IHD, comprising 32.3% of total CVD-related deaths. Although recent years have seen some improvements, women continue to experience slightly higher IHD-related mortality rates (29.9%) compared to men (27.6%).^5^ These statistics underscore the urgent need for targeted strategies to address gender inequities and the broader determinants of health in managing CVD.^6,7^

Adding to this complexity is the underrepresentation of women in studies assessing the outcomes of PCI, leaving the potential benefits for women underexplored and speculative. This lack of representation raises an unsettling question: are women’s coronary arteries receiving the attention they truly need? Like the legendary Hua Mulan, who disguised herself as a man to fight in the war, could current PCI guidelines—designed primarily with male patients in mind—overlook critical nuances in treating female patients? This metaphor serves as a poignant reminder that a one-size-fits-all approach may fail to address the unique challenges faced by women with coronary artery disease.

In this issue of the journal, Braga et al.^8^examine whether current guideline-oriented PCI provides Brazilian women the same benefits as their male counterparts. Conducted in a public tertiary cardiovascular center in Brazil, the study analyzed outcomes in 1,146 women who underwent PCI between 2019 and 2020. The cohort, with a mean age of 65 years, exhibited a high prevalence of traditional cardiovascular risk factors such as hypertension (88%), diabetes (47.5%), and dyslipidemia (85%). Most patients (69%) were admitted with acute coronary syndrome (ACS). PCI procedures were predominantly successful, with 97.7% of patients and 98.4% of treated vessels achieving favorable outcomes. Complications occurred in 14.2% of patients, with a 1.2% in-hospital mortality rate. Periprocedural myocardial infarction was reported in 3.6% of cases. However, the absence of data on the slow-flow/no-reflow phenomenon—a complication associated with poorer outcomes—limits the scope of understanding. Predictors of in-hospital major adverse cardiac and cerebrovascular events (MACCE) included prior stroke, chronic kidney disease, and procedural failure, highlighting the complex interplay of anatomical and clinical factors influencing outcomes in women.^9^

Despite these favorable outcomes, the study missed an opportunity to explore critical social determinants of health, such as educational levels and social support networks. These factors could offer deeper insights into their impact on cardiovascular health, and their exploration could lead to significant advancements in our understanding and management of CVD.

The study’s data, derived from a public hospital setting, present unique challenges, including a population characterized by lower socioeconomic and educational levels, limited use of advanced intravascular imaging and physiology tools, restricted availability of antiplatelet therapies, and cost-driven limitations on the types and numbers of drug-eluting stents used. These real-world conditions contrast sharply with the idealized settings in many international guidelines and clinical trials for treating acute or chronic coronary disease.^10-13^

Over an average of 576 days, follow-up data further confirmed the sustained benefits of PCI, with a MACCE-free survival rate of 86%, a 3.5% cardiac mortality, and 8% recurrent ACS, underscoring its effectiveness in managing acute and chronic coronary artery disease. Predictors of these events included admission for ACS at the index PCI and in-hospital MACCE, highlighting the importance of early intervention and comprehensive post-discharge management. These findings provide reassurance and hope for improving long-term outcomes for this high-risk population.

Finally, the answer is cautiously affirmative when addressing the “Hua-Mulan conundrum”—whether PCI evidence derived predominantly from male-focused studies can be applied to female patients. While this study corroborates significant progress in applying current guidelines for women with coronary artery disease, it also emphasizes the importance of tailoring approaches to account for women’s unique risk profiles, anatomical specificities, and social determinants of health.

Braga et al.^8^ powerfully remind us of both the progress made and the challenges in addressing cardiovascular health gender-related disparities. By prioritizing female-focused strategies, healthcare providers can improve outcomes for women with coronary artery disease, ensuring equitable access to care and enhancing survival rates. Collaborative research, education, and policy-making efforts will be pivotal in bridging these gaps and shaping a future where cardiovascular health outcomes may be less influenced by gender.
